# The Effect of a Low GI Diet on Truncal Fat Mass and Glycated Hemoglobin in South Indians with Type 2 Diabetes—A Single Centre Randomized Prospective Study

**DOI:** 10.3390/nu12010179

**Published:** 2020-01-08

**Authors:** Nivedita Pavithran, Harish Kumar, Arun Somasekharan Menon, Gopala Krishna Pillai, Karimassery Ramaiyer Sundaram, Omorogieva Ojo

**Affiliations:** 1 Department of Clinical Nutrition, Amrita Institute of Medical Sciences and Research Centre, Amrita VishwaVidyapeetham, Kochi, Kerala 682041, India; 2 Department of Endocrinology, Amrita Institute of Medical Sciences and Research Centre, Amrita VishwaVidyapeetham, Kochi, Kerala 682041, India; harishkumar@aims.amrita.edu (H.K.); arunsmenon@aims.amrita.edu (A.S.M.); 3 Department of General Medicine, Amrita Institute of Medical Sciences and Research Centre, Amrita VishwaVidyapeetham, Kochi, Kerala 682041, India; mgkpillai@aims.amrita.edu; 4 Department of Biostatistics, Amrita Institute of Medical Sciences and Research Centre, Amrita VishwaVidyapeetham, Kochi, Kerala 682041, India; krsundaram@aims.amrita.edu; 5 School of Health Sciences, University of Greenwich, London SE9 2UG, UK; o.ojo@greenwich.ac.uk

**Keywords:** low GI diet, truncal obesity, glycatedhemoglobin, anthropometric parameters, body composition indicators, body mass index

## Abstract

Background: There has been no previous study that has investigated the effect of a low glycemic index (LGI) diet with local recipes of South Indian cuisine on the body fat composition using dual-energy X-ray absorptiometry (DXA). Truncal obesity has been associated with the risk of metabolic disorders and cardiovascular diseases. Aim: The aim of this study was to examine the effect of a low GI diet on glycemic control and body composition in people with type 2 diabetes in South India. Method: This was a prospective and randomized controlled study that was conducted over a period of 24 weeks. A total of 40 participants were recruited from the Department of Endocrinology and Diabetes Outpatient in Kerala, South India. All the patients had type 2 diabetes and were randomly assigned and given advice and instructions to follow either a low GI diet plan (*n* = 18) or their usual diet, which served as control (*n* = 18). The advice was reinforced throughout the study period. Dietary compliance was evaluated based on a 24 h dietary recall at weeks 3, 11, 12, 18, 23, and 24. The age of the subjects ranged from 35 to 65 years. Anthropometric, body composition, and cardio-metabolic parameters were measured according to standard procedures. T-tests were conducted to compare differences between intervention and control groups and the Pearson correlation coefficient was used to evaluate associations between the variables. Results: There were significant reductions (*p* < 0.05) in the low GI diet compared to the control group with respect to weight, body mass index (BMI), and triceps skinfold thickness. Similarly, significant reductions were observed in the low GI diet group with respect to region, total fat, android, and gynoid fat mass and the differences between the groups were significant at *p* < 0.05. There was also a positive correlation between BMI and android fat mass (r = 0.745), total fat mass (r = 0.661), total truncal mass (r = 0.821), and truncal fat (r = 0.707). There was a significant reduction in glycated hemoglobin in the low GI diet group compared to the control group at *p* < 0.05. Conclusion: This study has demonstrated that there was a significant reduction (*p* < 0.05) of truncal obesity and glycated hemoglobin in patients with type 2 diabetes on a local diet of South Indian cuisine with low GI compared with the control.

## 1. Introduction

The burden of diabetes is on the rise across the globe, and the rising prevalence of type 2 diabetes, particularly in developing countries, appears to be mainly related to the increasing number of overweight and obese individuals [1].Among Asians, the prevalence of diabetes at any body mass index (BMI), waist circumference, and waist–to–hip ratio (WHR) is higher compared to the western population. The cut-off values in relation to these parameters for optimal discrimination of diabetes are lower in Asians compared with whites. Obesity is a strong risk factor for type 2 diabetes because of its association with insulin resistance. Overweight or obesity is seen in 30–65% of the adult urban Indians [2].Central (truncal) obesity has been found to associate more strongly with insulin resistance and with diabetes than generalized obesity [3,4,5].

Diabetes and obesity have emerged as a major public health problem and are increasing in rural and urban regions of India, particularly Kerala. The prevalence of obesity among people with diabetes in South Kerala is 66% [6]. The Amrita Diabetes and Endocrine Population Survey (ADEPS), a community-based cross-sectional survey, showed a prevalence of type 2 diabetes to be 9.0% in Central Kerala. The study also showed that obesity, abnormal subscapular triceps skinfold thickness, was found to be associated with an increased risk of diabetes mellitus (DM) [7].Epidemiological research generally uses anthropometric measures such as BMI and waist circumference (and sometimes waist-to-hip ratio) and truncal skinfold thicknesses as measures of obesity, as they are cheap to perform and universally available. However, when compared with advanced technologies, these are not as accurate in representing body fat and its distribution in specific regions. Hence in the past two decades, the development of body composition techniques such as dual-energy X-ray absorptiometry (DXA) has allowed more accurate quantification of ‘truncal’ fat.

Diet plays an important role in the management of type 2 diabetes. Dietary recommendations have emphasized the use of complex carbohydrates for slow digestion and absorption and will induce a slow postprandial glucose response. Of all the macronutrients, carbohydrates have the greatest effect on blood glucose and insulin levels, and its restriction has shown a significant impact on the management of diabetes [8].

Currently, India is undergoing a significant dietary transition from traditional diets to more ‘Western’ ways of eating and a concomitant epidemiological transition, which is associated with risk factors for nutrition-related non-communicable diseases (NCD) [9]. Whole-grains such as brown rice have been reported to have health benefits in reducing the risk of chronic diseases like diabetes. In addition, brown rice has an intact bran and a germ that contains micronutrients, phytonutrients, and dietary fiber as compared with fully polished white rice [10]. Low glycemic index diets cause a minimal increase in postprandial blood glucose levels compared to high GI foods [11]. They play a role in preventing type 2 diabetes and also in improving glycemic control in people with type 2 diabetes [12]. Unlike polished white rice, brown rice releases sugars slowly, thus helping to stabilize blood sugar in a sustained manner. This quality makes it a better option for people with diabetes mellitus. However, many people are unaware of the health benefits of brown rice and do not consider that Kerala red rice, parboiled rice, or even hand-pounded rice as brown rice.

There has been a nutrition transition in South India, which includes a substantial increase in refined carbohydrate intake at all three meals, and decreasing intake of whole-grain cereals, pulses, fruits, and vegetables [13]. Rice is the staple diet of South Indians. Although the glycemic index of white rice is high, other grains such as Kerala red rice, whole wheat, and millets are low in glycemic index. Furthermore, Rose matta rice or red rice is the most widely consumed rice variety in Kerala. Recently, the consumption of low GI ingredients of Kerala cuisine such as red rice and whole-grains are being replaced with white rice and commercially available wheat flour.

The effect of the consumption of locally available low glycemic index cereals of Kerala cuisine on glycemic control and body composition using DXA has not been examined previously. Several studies have used DXA to study fatness and fat distribution in persons with type 2 diabetes mellitus (T2DM) compared with healthy controls, but none have reported the changes in body composition with LGI recipes of Kerala cuisine. Therefore, the purpose of this study was to investigate the effect of a 24-week intervention with a low GI diet of Kerala cuisine on body composition, particularly truncal fat using DXA in people with type 2 diabetes mellitus. We hypothesized that an intervention of low GI diet using locally available ingredients of Kerala cuisine in South Indians would significantly decrease the fat mass, especially truncal fat mass values.

## 2. Methods

This was a prospective randomized controlled study conducted over a period of 24 weeks between October 2018 and April 2019 in the Department of Endocrinology and Diabetes Outpatient Department, Kerala, India.

Recruitment and study design: A randomized sampling technique was used to recruit the subjects. Every 3rd patient who visited the dietitian in the Department of Endocrinology and Diabetes OP was recruited and randomly assigned to either the interventional or control group.

Inclusion criteria: Subjects of both genders aged 35–65 years who had a history of diabetes for more than a year, with HbA1c ranging between 7–10%, and whose medications were unchanged for at least 3 months.

Exclusion criteria: Subjects who had a history of malabsorption, obese with BMI >35, physically handicapped, mental disorder or currently on antipsychotic medications, past/current history of cancer, chronic kidney diseases, congestive cardiac failure, chronic liver diseases, gastroparesis, or any other medically significant disease, any major surgeries for gastro-Intestinal diseases were excluded from the study.

Sample size: A pilot study was conducted to estimate the minimum sample size. Since there was no published paper in the existing literature with foods of Kerala cuisine and body composition in type 2 diabetes, a pilot study was done with 5 subjects in the low GI diet group and 5 subjects in the control group. Based on the main study variable, namely truncal fat, the mean ± S.D of the differences between baseline and 24 weeks in the LGI diet group was: −5.18 ± 5.61 and in the control group: 0.796 ± 0.306. Based on the results of the pilot study and with 80% power and 95% confidence, the minimum sample size was found to be 13 in each group. We recruited a total of 40 subjects with type 2 diabetes, 20 in each group.

### 2.1. Anthropometric Measurements

Height (in centimeters) was measured to the nearest of 0.1 cm by using a wall-mounted stadiometer. The individual was asked to stand upright without shoes with his/her back against the vertical backboard, heels together, and eyes directed forward. The weight (in kilograms) was measured using an electronic weighing scale that was kept on a firm horizontal flat surface. Subjects were asked to wear light clothing without shoes, and weight was recorded to the nearest 0.5 kg. Body mass index (BMI) was calculated as body weight (kg) divided by height (m^2^). The body mass index (BMI) was categorized into normal or lean (18.5–22.9 kg/m^2^), overweight (23.0–24.9 kg/m^2^), and obese (≥25kg/m^2^), based on the revised consensus guidelines for Asian Indians. Waist circumference (in centimeters) was measured using a non-stretchable measuring tape at the smallest horizontal girth between the costal margins and the iliac crest at the end of an expiration with an accuracy of 0.1 cm and the hip circumference was taken around the widest portions of the buttocks.

Waist circumference (WC) was categorized as normal and abnormal according to Asia Pacific Criteria by Misra et al. [2], and WC cut-offs of ≥90 cm in men and ≥80 cm in women have a high odds ratio for cardiovascular risk factors.

Biochemical analysis: A blood sample for fasting blood glucose was collected after 8–12 h of fasting in a grey vacutainer containing an anticoagulant and a stabilizer, i.e., EDTA and sodium fluoride and postprandial blood glucose sample was collected in another grey vacutainer 2 h after food. Whole blood was collected in a violet vacutainer coated with EDTA K2 to estimate glycated hemoglobin (HbA1c). The blood sample for analyzing fasting lipid profiles was collected using green vacutainer coated with lithium heparin, ammonium heparin, or sodium heparin. All samples were estimated using the Roche autoanalyzer at the central laboratory of the tertiary care hospital accredited by the National Accreditation Board of Testing and Calibration Laboratories (NABL). The interpretation for the blood glucose profile was considered according to ADA guidelines 2017 and the reference interval for lipids was considered as per National Cholesterol Education Program (NCEP) Adult Treatment Panel III Report.

Body composition measurement: The body composition was measured using DXA (enCORE-based X-ray Bone Desitometer). Body composition software measures the regional and whole-body lean and fat tissue mass and calculates other derivative values which can be displayed in user-defined statistical formats and trends and compared to reference populations. Prior to the scan, the height and weight of participants were measured. All attenuating materials (belts, metal buttons, etc.) were removed from the measurement region. The subject was made to lay in the center of the scanner table. The subject’s hands were turned on the sides with thumbs up, palms facing legs, and arms are alongside the patient’s body. The fat mass, which is composed strictly of fat, and the fat-free mass consist of everything in the body except fat, including organs, skin, and all body tissue such as muscle tissue were measured in kilograms. The percentage of body fat was calculated by dividing the total fat mass by total DXA mass (fat mass and fat-free mass) and multiplying by 100.

Android gynoid ratio: This is a measure that determines if the subject is “Apple” or “Pear” shape but does not tell you if that mass is fat or lean. It is strictly a ratio of the percentage fat of android and the percentage fat of gynoid. If the subject has pear habitus, then there is less risk for cardiovascular problems. The android region included the area between the pelvis cut line extending upwards to include 20% of the distance between the pelvis and neck cut lines. The gynoid region was positioned with the upper boundary positioned below the cut line at a distance equal to 1.5 times the height of the android region. The lower boundary was located a distance 3.5 times the height of the android region from the pelvis cut line.

Fat mass index and fat-free mass index: FMI and FFMI were calculated by dividing fat mass (FM) and fat-free mass (FFM) by the square of height.

Dietary intervention: All subjects enrolled in the study were interviewed for their home recall and 24 h dietary recall to assess their dietary habits and cultural practices. Three components were assessed during the interview: Meal timing, quality, and quantity of diet. Meal timings were classified into proper and improper meal timings. The quality of the diet was assessed using a detailed food frequency questionnaire (FFQ) consisting of 59 items. The FFQ included commonly consumed food items in Kerala from all food groups and also included bakery items, soft drinks, and miscellaneous foods. Intake of any weight reducing commercial supplements or medications were also collected. Subjects on any of these weight reducing formulas were excluded.

The control subjects were advised and given instructions to consume a regular diet, whereas the intervention group was advised and given instructions to include whole-grain cereals over the study period. This advice was reinforced by the dietitian throughout the study. The diet plan (Table 1) in the low glycemic index (LGI) group included foods that were low in GI and were traditionally used by Kerala population.

Dietary Compliance: Dietary compliance was evaluated by obtaining a 24h dietary recall at weeks 3, 11, 12, 18, 23, and 24. The compliance was checked by dieticians during the telephonic interview. During the interview, the foods consumed by the subject in the last 24 h were recalled and noted.

The calorie intake of both groups was matched during the study. This was ensured by calculating the 24 h recall during the follow-up.

This study was approved by the Institutional Ethics Committee of Amrita Institute of Medical Sciences and Research Centre (No. IEC-AIMS-2018-DIET-165) and registered under Clinical Trials Registry-India (ICMR-NIMS) CTRI Reg No.: CTRI/2019/12/022425.

### 2.2. Statistical Analysis

Descriptive analyses were used to describe the study sample, and chi-square and/or *t*-test analysis was used to compare the sociodemographic characteristics of the study population in this study. Differences in demographic characteristics were calculated using the χ^2^ test for categorical variables and nonparametric Wilcoxon test for continuous variables. Paired sample Student’s *t*-test was used to compare the baseline and week 24 within the intervention group and the control group. The independent Student’s t-test was used to compare differences between the intervention and control groups. The mean difference between the pre-post/SD difference for all variables was also calculated. The Pearson correlation coefficient was used to find associations between the study variables. Statistical analysis was performed using IBM SPSS 20.0 for Windows (SPSS Inc., Chicago, IL, USA). All the statistical tests were based on the two-tailed hypothesis and the significance level was defined as *p* < 0.05.

Descriptive analyses were used to describe the study sample, and chi-square and/or *t*-test analysis was used to compare the sociodemographic characteristics of the study population in this study. Differences in demographic characteristics were calculated using the χ^2^ test for categorical variables and nonparametric Wilcoxon test for continuous variables. Paired sample Student’s *t*-test was used to compare the baseline and week 24 within the intervention group and the control group. The independent Student’s t-test was used to compare differences between the intervention and control groups. The mean difference between the pre-post/SD difference for all variables was also calculated. The Pearson correlation coefficient was used to find associations between the study variables. Statistical analysis was performed using IBM SPSS 20.0 for Windows (SPSS Inc., Chicago, IL, USA). All the statistical tests were based on the two-tailed hypothesis and the significance level was defined as *p* < 0.05.

## 3. Results

A total of 36 subjects (LGI = 18; control = 18) completed the study. The mean age of the study subjects was 52 ± 7.7 years. In the LGI group, 50% were females and the remaining 50% were males. In the control group, 67% were males and the females represented 33%. The mean duration of diabetes was 7.48 ± 2.7 years in the LGI group vs. 7.45 ± 2.5 years in the control group. The variables such as weight, BMI, body fat composition, and metabolic variables were comparable at baseline between the groups as shown in Table 2. In addition, there were no differences observed with respect to gender in the study variables between the groups.

Anthropometric variables of the subjects: Anthropometric measurements such as weight, BMI, waist circumference, and triceps skinfold thickness were compared at baseline and 24 weeks between the LGI and control groups. After 24 weeks of intervention, body weight decreased in the LGI group as shown in Table 3. However, the waist circumference did not change in the groups after intervention. There was a significant reduction of TSF in the LGI group and these changes were statistically significant at *p* < 0.001 between the groups.

### 3.1. Biochemical Profile of Study Subjects

The low glycemic index recipes of the Kerala cuisine brought a significant reduction in HbA1c by 0.8% in the intervention group and an increase of 0.24% in the control group. The difference between the intervention and control groups with respect to HbA1c was statistically significant (*p* = 0.000) (Table 4). In addition, the lipid profile in the LGI group showed statistical significance post-intervention in total cholesterol, triglycerides, and very-low-density lipoprotein (VLDL).In the control group, there was an increase in low-density lipoprotein (LDL) by 1.63mg/dL. Although serum triglycerides were within normal Fin both groups at baseline, after 24 weeks of intervention, the LGI group did show a reduction, which was statistically significant. However, high-density lipoprotein, which is the good cholesterol, wasin the normal range at baseline and remained unchanged in both groups.

### 3.2. Body Composition Variables Using DXA

The body composition measurements by DXA showed a 0.93% reduction in regional fat in the LGI group and an increase in fat percent in the control group (Table 5). There was a significant reduction at *p* < 0.001 in regional fat between the two groups. The truncal fat, which is the main component in reducing lifestyle diseases, had shown a significant reduction in the intervention group compared to the control group with *p* < 0.001. However, there was no change in the fat-free mass and A/G ratio in the subjects.

**Figure 1:**
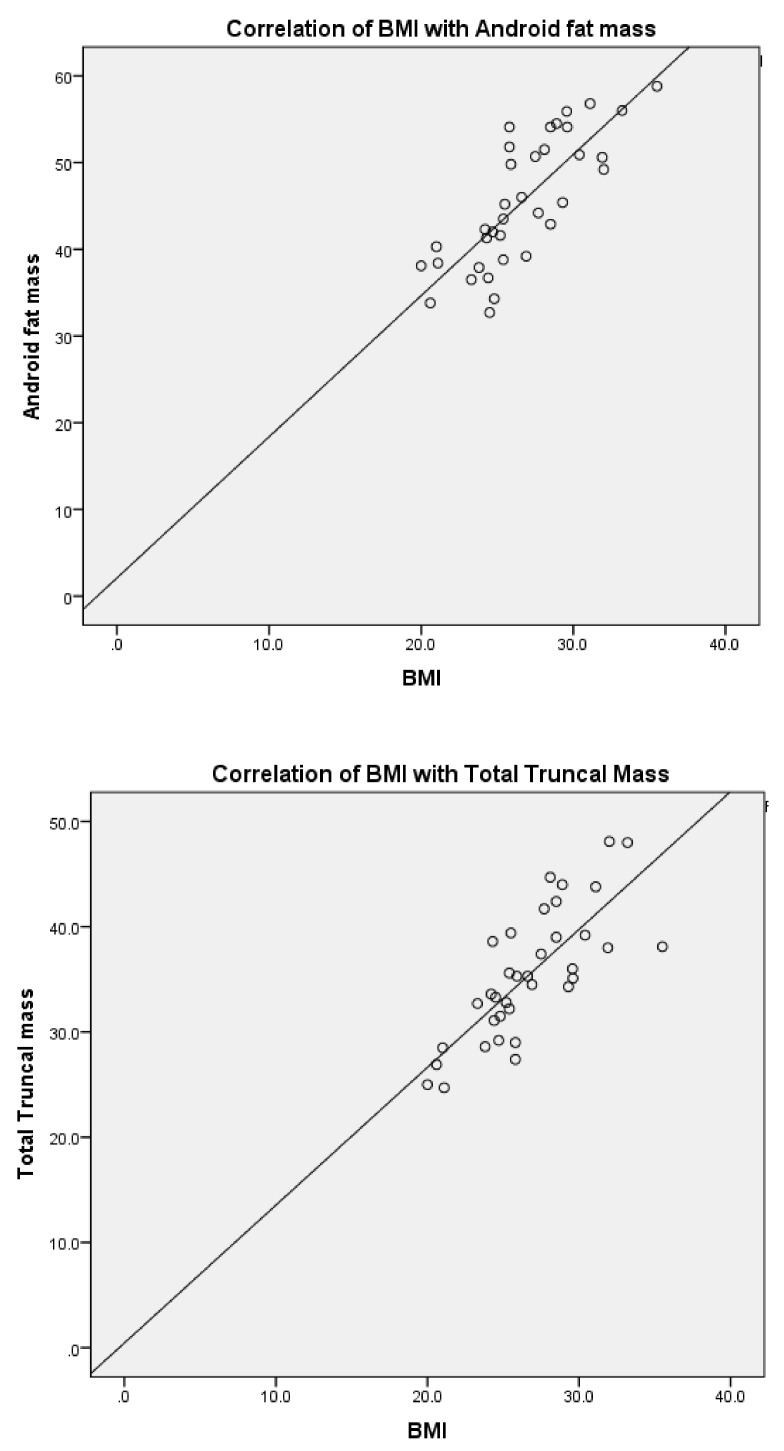

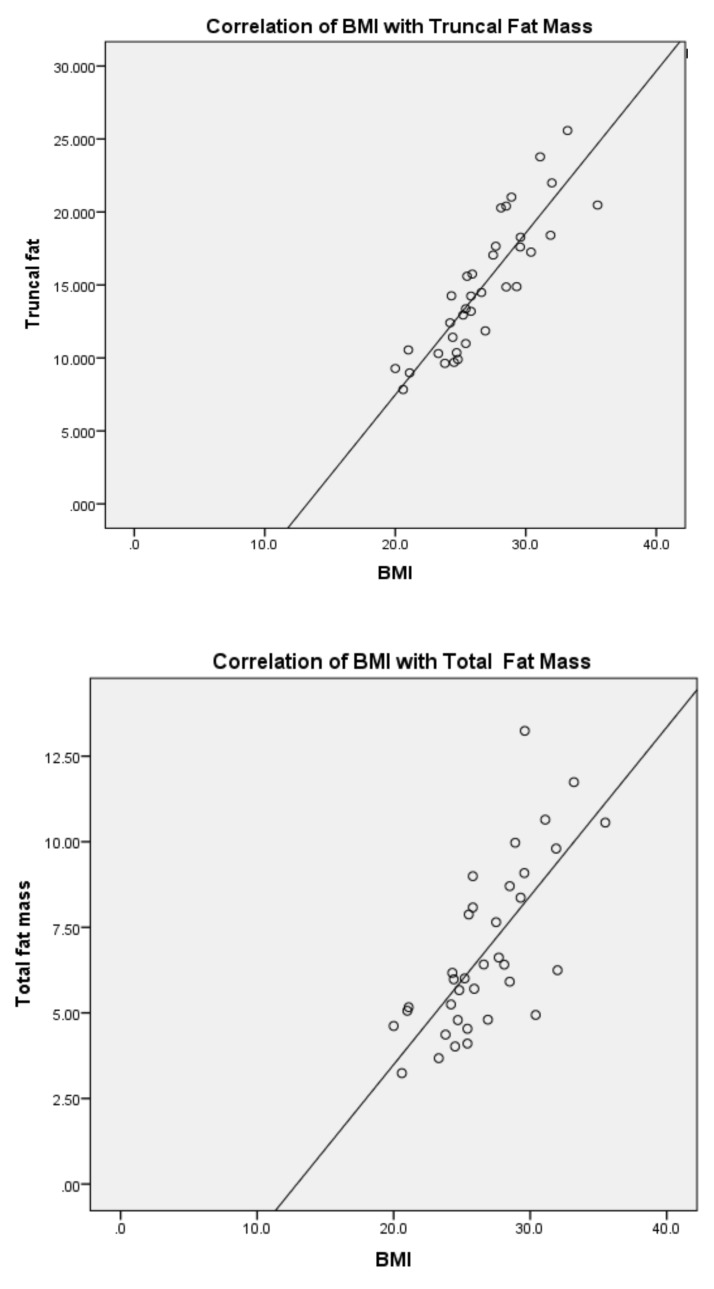
Showing the correlation between BMI, android fat, total fat mass, total truncal mass, and truncal fat in people with type 2 diabetes.

Correlations: The correlation between BMI and body composition is shown in Figure 1. There was a strong positive correlation between BMI and android fat (r = 0.745)**, total fat mass (r = 0.661)**, total truncal mass (r = 0.821)** and truncal fat (r = 0.707)**.There was a positive correlation between waist circumference and body composition variables at *p* < 0.01, as shown in Table 6. However, there was no correlation between gynoid fat mass and BMI. The waist circumference also did not show any correlationwith gynoid fat mass.

## 4. Discussion

This is the first study to report the effect of local recipes of Kerala cuisine, which is low in GI, on truncal fat and body fat variables in people with type 2 diabetes. The nutritive value and health benefits of low GI recipes like the use of Kerala red rice and whole wheat have been documented, but its long term effect on the glycemic and lipid profile have never been extensively studied. In our study, the use of Kerala red rice and broken wheat showed significant changes in glycemic and body fat composition in people with type 2 diabetes. The red rice’s high-fiber content caused weight loss, which was likely the major contributor to the improvement in glycated hemoglobin. The red rice’s high fiber content could also have contributed to the improvement in glycated hemoglobin through slowed absorption of carbohydrates. Other characteristics of the red rice that could have contributed to the improvement in the glycated hemoglobin include the red rice’s high content of Mg and Ca, which have been found to reduce insulin resistance, and the red rice’s high content of proanthocyanidins, which have been shown to provide protection against type 2 diabetes [14,15]. Furthermore, there is evidence that dietary patterns of whole-grain consumption have reduced the risk of type 2 diabetes [16]. In our study, 2–3 servings of whole-grains had reduced the glycemic and body fat composition. Previous studies have shown similar results where the risk of type 2 diabetes was reduced by 20–30% in subjects who consumed 2-3 servings of whole-grains [17,18,19,20].

A study conducted by Gomes [21] showed that consuming a low GI diet for 30 days in overweight patients with type 2 diabetes caused a significant reduction of approximately 2% in body fat. In the present study, there was a reduction of 2.9% body fat in the LGI group, which showed a greater reduction in our study population compared to the above study.

In this study, there was a significant reduction in HbA1c and a reduction in weight and subcutaneous fat in the LGI group after 6 months. In a randomized crossover study, 19 women, with excess body weight (BMI between 25 and 47 kg/m^2^), aged between 34 and 65 years old, were advised to include in at least 3 of their daily meals high GI or low GI foods. After 12 weeks, there was no difference in energy intake, body weight, and WC in response to the study treatments [22]. On the other hand, a European study showed that consumption of a high GI diet resulted in an increase in WC and BMI [23]. A similar study on the consumption of a low GI diet for 6 months showed a significant decrease in HbA1c, although there was an increase in body weight. However, WC, HC, and BMI remained unchanged, thus confirming the results of the previous study [24]. Such results suggest that the intake of a low GI diet can decrease the occurrence of obesity, including visceral obesity. In a cross-over study done by Bouche et al. [25], high and low GI diets were consumed by nondiabetic men during a period of 5 weeks. They observed a significant reduction in the adiposity in overweight men who consumed low GI diets [25]. Another study by Costa [26] showed that the consumption of 2 daily low GI meals for 30 consecutive days led to a significant reduction in WC and WHR in subjects with excessive body weight. However, energy intake, body weight, and body fat were not affected. They concluded that the consumption of low GI diets could be useful in decreasing and controlling abdominal obesity [26].

Dietary management approaches for improved glycemic control and weight loss in patients with type 2 diabetes may depend on the use of diets with a low glycemic index instead of using a standard low-fat diet [27]. A systematic review done by Thomas and Elliot suggested that lowering the glycemic index of food may improve glycated hemoglobin in patients with diabetes. They concluded that foods with a low GI might contribute to glycemic control compared to foods with a high GI, through the promotion of insulin sensitivity, reducing fluctuations in blood glucose levels and reducing daily insulin requirements [28]. Our study showed a significant reduction in HbA1c values by 0.8% with low GI preparations compared to their regular diet. However, in another study that compared the low glycemic index diet and standard diabetic diet, there was no statistically significant difference between the low GI and higher GI diets in relation to HbA1c and fasting blood glucose [29]. A study done by Yusof et al. [30] showed that although the effect on HbA1c was not significantly different between the low-GI diet and the higher-GI diet or control, the improvement within the low-GI group was more pronounced and of clinical benefit. A study done by David et al. [31] showed that a 6-month treatment with a low-glycemic index diet in patients with type 2 diabetes resulted in moderately lower HbA1c levels compared with a high-cereal fiber diet.

Some studies demonstrated a significant impact of low-GI diets on blood lipids. They suggest that low-GI diets could have a positive effect on the blood lipid profile, especially for total cholesterol and LDL cholesterol [32]. However, our study did not show any changes in the lipid profile after intervention due to a normal lipid profile at baseline. Inclusion of a large randomized controlled trial with elevated lipids at baseline will be able to help us understand the full effect of a low-GI diet on lipids.

The Look AHEAD (Action for Health in Diabetes) study showed that those who lost 5% to <10% ([means ± SD] 7.25 ± 2.1 kg) of their body weight had increased odds of achieving a 0.5% point reduction in HbA1c (odds ratio 3.52 [95% CI 2.81–4.40]), a 5mmHg decrease in diastolic blood pressure (1.48 [1.20–1.82]), a 5mmHg decrease in systolic blood pressure (1.56 [1.27–1.91]), a 5 mg/dL increase in HDL cholesterol (1.69 [1.37–2.07]), and a 40 mg/dL decrease in triglycerides (2.20 [1.71–2.83]) [33]. Our findings showed that with a 2.5% reduction in weight, there was 0.8% reduction in HbA1c indicating that the LGI preparations of the Kerala cuisine was more effective in glycemic control as well as in reducing the cardiovascular risk associated with type 2 diabetes.

Several studies used dual-energy X-ray absorptiometry, as this is considered the gold standard in measuring total body composition and fat content with a high degree of accuracy. The scan is highly accurate compared with most other methods for determining body composition and highly useful for tracking change in muscle and fat over time. The present study showed a significant reduction of 2.9% regional fat and 5.2% reduction in total fat mass after 6 months of intervention with LGI compared to their usual diets. This reduction is desirable, especially among patients with type 2 diabetes, since body fat is positively correlated with cardiovascular disease risk.

Truncal obesity (android and gynoid fat) has been associated with cardiovascular disease risk in type 2 diabetes. The present study showed a significant reduction in android fat and an increase in gynoid fat, which is considered to be cardioprotective.

Studies conducted in various countries around the world show that adherence to dietary recommendations is poor among patients with diabetes. They showed that patients with diabetes have difficulty implementing recommended dietary changes in their daily routines and lifestyle [34,35,36]. In diabetes populations, a person will be better able to follow the prescribed diet when the foods specific for diabetes are acceptable culturally, socially, and personally. A study conducted by Lawton et al. [37] showed that individuals with diabetes had difficulty altering their food habits and often tended to consume traditional foods that were high in fat and sugar. Another study examining diabetes dietary satisfaction also found improved A1c when participants expressed better ability to implement a diabetes diet, including with regard to social situations, but cultural considerations were not addressed [38].When optimizing dietary patterns in order to manage diseases, the food culture of the society and the individual should be considered in order to maximize the acceptability of the treatment. Choosing unfamiliar foods from a different ethnic heritage might make dietary adherence to diabetes guidelines more complicated and could contribute to low adherence rates, while the acceptability of a recommended diet could increase adherence. Therefore, understanding the personal and cultural barriers that are associated with dietary adherence faced by people with diabetes could contribute to a better intervention program. This was observed in the current study as the recipes recommended to the low GI group were traditionally consumed by the population. Hence the compliance and acceptability to the diet was much higher in the intervention group.

In the present study, the diets were well tolerated and apparently there were no food intolerances or side effects. The advantage of our study was the inclusion of local recipes that were commonly consumed by the population to enhance adherence to the prescribed diet. These strategies helped to improve the intake, and overall compliance was excellent. The local recipes also provided satiety, which is very crucial in the management of type 2 diabetes.

## 5. Limitations of the Study

The small sample size of our study limited the statistical power to conduct a multivariate statistical analysis. However, the randomization process was carefully conducted by us. Because of that, the control group and LGI group presented similar baseline body composition, besides clinical, biochemical, and anthropometric data.

## 6. Conclusions

This study has shown that there was a significant reduction (*p* < 0.05) of truncal obesity and glycated hemoglobin in patients with type 2 diabetes on low GI diet compared with control. The reduction in weight and improvement in HbA1c with supplementation of low GI diet over a 24 week period has shown a beneficial effect in reducing the metabolic and CVD risk. Thus, this local diet of South Indian cuisine with low GI has the potential to reduce truncal fat and weight, and promote glycemic control in patients with type 2 diabetes.

## Figures and Tables

**Table 1:** The diet plan for the low glycemic index (GI) diet group.

Breakfast	Lunch	Dinner
Red rice puttu (GI = 38) Whole wheat flour (GI = 45) puttu Barley (GI = 25) puttu Rolled oats/Steel cut oats puttu (GI = 51/52)	Rose Matta rice (GI = 38)	Broken wheat (GI = 41) upma Broken wheat + green gram+ fenugreek seeds kanji (porridge) Whole wheat flour (GI = 45) roti

**Table 2:** Baseline characteristics of the study subjects.

Parameters	LGI Group (*n =* 18)	Control Group (*n =* 18)	*p* Value
Weight(kg)	67.91 ± 12.56	73.12 ± 8.76	0.156
BMI	26.81 ± 5.04	27.25 ± 2.72	0.266
Waist circumference	93.17 ± 11.18	96.77 ± 10.72	0.213
TSF	26.72 ± 4.45	23.31 ± 5.50	0.104
Total fat %	37.01 ± 7.10	34.8 ± 7.13	0.295
Total fat mass (g)	25,499.61 ± 8442.76	25,768.94 ± 7389.81	0.896
Truncal fat (g)	14,941.94 ± 4644.29	15,204.61 ± 4755.44	0.809
Lean mass (g)	40,207.89 ± 6747.26	44,924.61 ± 4972.96	0.021
Fat free mass(g)	42,430.94 ± 7105.78	47,339.00 ± 5222.62	0.179
Android(%fat)	47.11 ± 6.83	45.05 ± 7.39	0.432
Gynoid(% fat)	37.81 ± 9.49	35.23 ± 8.53	0.359
A/G ratio	1.29 ± 0.22	1.31 ± 0.20	0.395

Abbreviations: BMI (body mass index); TSF (triceps skinfold thickness): A/G ratio (android-gynoid-ratio); LGI (low GI).

**Table 3:** Comparison of differences between baseline and 24 weeks in Anthropometric measurements between the LGI and Control groups.

Variable	Group	Baseline (Mean ± S.D)	24 Week (Mean ± S.D)	Mean Change	*p* Value
Weight	Control	73.12 ± 8.76	73.40 ± 9.03	0.28 ± 1.48	0.007 *
	LGI	67.91 ± 12.56	66.02 ± 11.05	−1.88 ± 2.85	
BMI	Control	27.25 ± 2.72	27.32 ± 2.78	0.07 ± 0.56	0.014 *
	LGI	26.81 ± 5.04	26.06 ± 4.23	−0.75 ± 1.23	
Waist circumference	Control	96.77 ± 10.72	94.39 ± 13.41	−2.38 ± 6.38	0.584
	LGI	93.17 ± 11.18	89.81 ± 10.67	−3.37 ± 4.02	
Triceps skinfold	Control	23.31 ± 5.50	22.56 ± 7.17	−0.75 ± 4.93	0.001 *
	LGI	26.72 ± 4.45	20.17 ± 5.22	−6.55 ± 5.10	

Abbreviation: BMI (Body mass index); LGI (Low GI); * Shows significant differences

**Table 4:** Metabolic parameters of low GI and control diets in people with type 2 diabetes.

Variable	Diet	Baseline (Mean ± SD)	Follow-Up (Mean ± SD)	*p* Value
**HbA1c (%)**	Control	8.18 ± 0.98	8.42 ± 1.16	0.001 **
	LGI	8.28 ± 0.91	7.41 ± 0.89	
**Total cholesterol (mg/dL)**	Control	154.08 ± 34.11	155.08 ± 38.89	0.116
	LGI	176.43 ± 38.75	158.98 ± 31.91	
**Triglycerides (mg/dL)**	Control	132.61 ± 70.12	129.50 ± 51.02	0.24
	LGI	127.95 ± 41.35	115.04 ± 33.14	
**HDL (mg/dL)**	Control	39.49 ± 11.85	39.93 ± 10.96	0.80
	LGI	47.53 ± 13.97	47.48 ± 12.05	
**LDL (mg/dL)**	Control	102.17 ± 31.45	103.81 ± 35.79	0.233
	LGI	119.12 ± 33.81	108.03 ± 25.41	
**VLDL (mg/dL)**	Control	26.51 ± 14.04	25.89 ± 10.20	0.346
	LGI	25.31 ± 8.1	22.06 ± 6.20	

Abbreviations: HbA1c (glycated hemoglobin); HDL (high-density lipoprotein); LDL (low-density lipoprotein); VLDL (very-low-density lipoprotein); LGI (Low GI). ** highly significant at *p*-value < 0.01 level.

**Table 5:** Comparison of differences between baseline and 24 weeks in body composition measurements between the LGI and control groups

Variable	Group	Baseline (Mean ± S.D)	24 Week (Mean ± S.D)	Mean Difference	*p* Value
Region (% fat)	Control	34.8 ± 7.13	35.57 ± 6.80	0.77 ± 1.23	0.001 **
	LGI	37.01 ± 7.10	36.07 ± 7.68	−0.93 ± 1.42	
Truncal fat (g)	Control	25,768.94 ± 7389.81	26,413.06 ± 7292.36	644.12 ± 1.32	0.001 **
	LGI	25,499.61 ± 8442.76	24,116.72 ± 7668.37	−1382.9 ± 1.85	
Lean mass (g)	Control	44,924.61 ± 4972.96	44,553.28 ± 5029.57	−371.3 ± 0.92	0.815
	LGI	40,207.89 ± 6747.26	39,748.39 ± 6658.78	−459.5 ± 1.28	
Fat free mass (g)	Control	47,339.00 ± 5222.62	46,960.78 ± 5324.87	−378.2 ± 0.90	0.808
	LGI	42,430.94 ± 7105.78	41,963.17 ± 7065.33	−467.8 ± 1.26	
Android (% fat)	Control	45.05 ± 7.39	45.78 ± 7.16	0.72 ± 2.47	0.010 **
	LGI	47.11 ± 6.83	45.33 ± 7.93	−1.78 ± 2.99	
Gynoid (%fat)	Control	35.23 ± 8.53	36.44 ± 8.49	1.21 ± 1.71	0.009 **
	LGI	37.81 ± 9.49	37.31 ± 9.53	−0.50 ± 2.01	
A/G ratio	Control	1.31 ± 0.20	1.28 ± 0.19	−0.02 ± 0.11	0.672
	LGI	1.29 ± 0.22	1.25 ± 0.20	−0.03 ± 0.07	

Abbreviations: A/G ratio (Android-gynoid-ratio). ** highly significant at *p*-value < 0.01 level.

**Table 6:** Correlation between waist circumference and body composition variables in low GI and control diets in people with type 2 diabetes.

Variables	Waist Circumference	*p* Value
	Pearson Correlation Coefficient (r)	
Android (% fat)	0.54 **	<0.01
Total mass	0.705 **	<0.01
Total fat mass	0.344 *	<0.05
Total lean mass	0.635 **	<0.01
Total truncal mass	0.779 **	<0.01
Truncal fat	0.710 **	<0.01
Truncal lean	0.511 **	<0.01

** Correlation is significant at the 0.01 level (2 tailed).
